# Exploring the biochemical dynamics in faba bean (*Vicia faba* L. minor) in response to *Orobanche foetida* Poir. parasitism under inoculation with different rhizobia strains

**DOI:** 10.1371/journal.pone.0304673

**Published:** 2024-05-31

**Authors:** Manel Bouraoui, Zouhaier Abbes, Boulbaba L’taief, Mohammed O. Alshaharni, Neila Abdi, Amira Hachana, Bouaziz Sifi

**Affiliations:** 1 Field Crops Laboratory, National Institute for Agricultural Research of Tunisia (INRAT), Carthage University, Tunis, Tunisia; 2 Sciences Faculty of Bizerte (FSB), Carthage University, Tunis, Tunisia; 3 Biology Department, College of Sciences in Abha, King Khalid University, Abha, Saudi Arabia; 4 Department of Plant Sciences (Plant Breeding), University of the Free State, Bloemfontein, South Africa; 5 Agronomic Sciences and Technology Laboratory, National Institute for Agricultural Research of Tunisia (INRAT), Carthage University, Tunis, Tunisia; Universidade Federal de Minas Gerais, BRAZIL

## Abstract

In Tunisia, *Orobanche foetida* Poir. is considered an important agricultural biotic constraint on faba bean (*Vicia faba* L.) production. An innovative control method for managing this weed in faba bean is induced resistance through inoculation by rhizobia strains. In this study, we explored the biochemical dynamics in *V*. *faba* L. minor inoculated by rhizobia in response to *O*. *foetida* parasitism. A systemic induced resistant reaction was evaluated through an assay of peroxidase (POX), polyphenol oxidase (PPO) and phenyl alanine ammonialyase (PAL) activity and phenolic compound and hydrogen peroxide (H_2_O_2)_ accumulation in faba bean plants infested with *O*. *foetida* and inoculated with rhizobia. Two rhizobia strains (Mat, Bj1) and a susceptible variety of cultivar Badi were used in a co-culture Petri dish experiment. We found that Mat inoculation significantly decreased *O*. *foetida* germination and the number of tubercles on the faba bean roots by 87% and 88%, respectively. Following Bj1 inoculation, significant decreases were only observed in *O*. *foetida* germination (62%). In addition, Mat and Bj1 inoculation induced a delay in tubercle formation (two weeks) and necrosis in the attached tubercles (12.50% and 4.16%, respectively) compared to the infested control. The resistance of *V*. *faba* to *O*. *foetida* following Mat strain inoculation was mainly associated with a relatively more efficient enzymatic antioxidative response. The antioxidant enzyme activity was enhanced following Mat inoculation of the infected faba bean plant. Indeed, increases of 45%, 67% and 86% were recorded in the POX, PPO and PAL activity, respectively. Improvements of 56% and 12% were also observed in the soluble phenolic and H_2_O_2_ contents. Regarding inoculation with the Bj1 strain, significant increases were only observed in soluble phenolic and H_2_O_2_ contents and PPO activity (especially at 45 days after inoculation) compared to the infested control. These results imply that inoculation with the rhizobia strains (especially Mat) induced resistance and could bio-protect *V*. *faba* against *O*. *foetida* parasitism by inducing systemic resistance, although complete protectionwas not achieved by rhizobia inoculation. The Mat strain could be used as a potential candidate for the development of an integrated method for controlling *O*. *foetida* parasitism in faba bean.

## Introduction

Faba bean (*Vicia faba* L.) is one of the major legume crops in the world [1]. It is the fourth most important pulse crop after chickpeas, dry peas, and dry beans [2]. It is widely grown and broadly cultivated in many regions of the world [3]. In Tunisia, faba beans are the major grain occupying 75% of the total grain legume area [4]. However, crop production and the cultivated area fluctuate due to several abiotic and biotic stresses. For example, broomrapes (*Orobanche*) are biotic constraints that cause considerable damage to faba bean in Tunisia [5]. The two major *Orobanche* species are *O*. *foetida* and *O*. *crenata* in Tunisia [6]. *O*. *foetida* is largely distributed in the western Mediterranean area where infects wild legumes. This constraint has also been reported to affect common vetch in Morocco [7] and faba bean in Tunisia [6]. In the Beja region of Tunisia, *O*. *foetida* has been recognised as a major agricultural parasite in faba bean [8]. This species is able to develop mainly on broad beans, lentils and vetch with variable levels of parasitism [9]. Several control strategies for managing *Orobanche* parasitism, from chemical control to cultural practices, have been employed and proposed [10–12]. However, complete protection has not been achieved by any one of these control strategies. Herbicides cannot differenciate between parasitic plants and crops [7] an interest has increased in alternative potential sustainable strategies as biological control. This can be used as effective alternative in eco-friendly agriculture and sustainable environments. According to Fernández-Aparicio et al. [13], the biological control of broomrape (*Orobanche*) depends on living organisms that are used to either eliminate seed banks or interfere with its ability to recognise hosts. For this purpose, phytophagous insects, mycoherbicides and bacteria have been employed. Phytophagous insects, such as *Phytomyza orobanchia* have been used to prevent the build-up of broomrape seed banks. *Fusarium* sp. or *Ulocladium botrytis* have been employed as mycoherbicide agents to attack broomrape seeds and radicles. Bacteria, such as *Pseudomonas aeruginosa*, *P*. *fluoresens* and *Bacillus subtilis* have also been employed as biocontrol agents. Like mycorrhizal fungi and rhizobia, they interfere with the broomrape’s ability to recognise its host. Indeed, where crop roots have been colonized by arbuscular mycorrhizal fungi and rhizobia, broomrape seeds have been found to be less capable of recognising the crop roots [13]. A combination of agronomic, chemical and biocontrol approaches would be the most appropriate approach for an integrated *Orobanche* management program. For sustainable agricultural development, Arfaoui et al. [14] suggested that rhizobia can be used as a biocontrol agent. It has been found that symbiosis with rhizobia strains could result in the better development and lower susceptibility of chickpea and pea to *O*. *crenata* [15, 16]. Bouraoui et al. [17] determined that using rhizobia strain inoculation in faba bean can bio-protect it from *O*. *foetida* parasitism and enhance faba bean productivity and nitrogen (N_2_) fixing. These rhizobia known as N_2_-fixing plant growth-promoting rhizobacteria (PGPR) and have been used as a biofertiliser due to their exceptional quality of being able to establish functional symbiosis with legumes. The PGPR (rhizobia) act directly and indirectly to establish this useful interaction. Directly, rhizobia help in fixing atmospheric N_2_ and facilitating the N_2_ supply to the plants, which is one of the major nutrient elements. In addition, they promote nutritional processes by solubilising several essential minerals, including phosphate solubilisation and production of siderophore. Moreover, they synthesis plant hormones such as indole-3-acetic acid and gibberellic acid [18, 19]. Indirectly, rhizobia elicit plant defence reactions against phytopathogens [18, 20, 21]. Through this antagonistic activity, they also stimulate plant growth by protecting the plant against fungal pathogens [21, 22] and *Orobanche* parasitism [17, 23, 24]. Other indirect mechanisms include microbial plant growth promoters, which are involved in different processes, such as the production of secondary metabolites that possess antibiotic qualities or produce antifungal substances, insecticides and immune-suppressants and stimulators of the plant’s defence system that eliminates attack by phytopathogens [18, 25, 26]. In addition to that, the PGPB rhizobia can play a role in protecting plants against *Orobanche* infection [17, 23, 24, 27] through the activation of phenylpropanoid activities and antioxidant mechanisms when plants respond to biotic stress [28]. This induced resistance results from the interaction of a suitable inducing agent with a plant [29]. When induced by eliciting agents that may be of microbial origin and/or during the pathogen-host interaction involving biocontrol agents, for example, innate immune resistance can be triggered [30–32].

During a plant’s response to stress, an induced defence mechanism can be installed that promotes several non-enzymatic components, based on flavonoids, phenolic components, enzymes, antioxidant enzymes (e.g. superoxide dismutase, peroxidases [POXs], catalase and glutathione reductase) and lignins for phenol mechanisms, such as polyphenol oxidase (PPO) and phenylalanine ammonia-lyase (PAL) [32–35].

The biotic elicitor (e.g. algae, fungi, bacteria and plant extracts) plays a major role in controlling diseases through induced resistance [36–40]. Rhizobia have been used when dealing with bacteria in bio-fertilization [41–43], broomrape parasitism management [15–17, 37] and biological pathogen control [44].

In this study, we explored the biochemical dynamics in faba bean inoculated with two rhizobia strains in response to *O*. *foetida* parasitism. It was hypothesized that rhizobia inoculation would induce resistance to *O*. *foetida* parasitism in the faba bean through the expression of several defence enzymes (POX, PPO and PAL) and the accumulation of phenolic compounds and hydrogen peroxide (H_2_O_2_) contents. This study was aimed at contributing to our understanding of the specific mechanisms by which rhizobia inoculation induces resistance to broomrape in faba bean.

## Materials and methods

### Biological material and trial layout

The rhizobia strains and faba bean seeds were donated by the Field Crop Laboratory and Agronomic Science Technology laboratory at National Institute of Agricultural Research in Tunisia. The faba bean variety (cv. Badi) used in this work is known for its excessive productivity and its susceptibility to *O*. *crenata* and *O*. *foetida* [45]. Broomrape seeds (*O*. *foetida*) were collected from flowering parasites on faba bean during the cropping season of 2013–2014 from the Beja region in north-western Tunisia (36°43’32”N, 9°10’54”E; altitude above sea level = 248 m). The seeds were cleaned in 2% calcium hypochlorite, for 5 minutes and then rinsed five times with sterile water. The rhizobia (strains Mat and Bj1) were collected from the Bizerte and Beja regions, respectively, and purified. The choice of these two strains was based on previously published research [17, 27]. The Mat strain was selected due to its antagonist effect on *O*. *foetida* and its high N_2_-fixing capacity. The Bj1 is one of the most effective rhizobia strains and was used in a nodulation test [17, 27]. Both strains were grown at 28°C on a yeast extract mannitol medium containing 1% mannitol (w/v) and 0.1% yeast extract (w/v). Stocks of the strains were also prepared on yeast extract-mannitol agar and kept at 4°C as source cultures [46]. A culture was prepared every six months in order to have stocks of younger generations.

### Evaluation of rhizobia as resistance inducers against *Orobanche foetida*

#### Co-culture using Petri dish experiments

Following Bouraoui et al. [27] co-culturing was performed using plastic Petri dishes (120 x120 x17 mm, Greiner). Three holes were made in each dish. A large hole was made in a large board to allow the faba bean shoots to grow through, and two others holes were drilled through the lower rim of the plastic Petri dishes for the plant roots fitting. The Petri dishes were filled with purified sand moistened with 50 ml of purified water and then covered with a wetted filter paper. Seeds of purified *Orobanche* (15 mg) were expanded between the fiberglass filter paper and the dish cover. These were left at 21°C for 7 days in a vertical position in purified water in a tray of purified polypropylene covered with aluminium foil.

The faba bean (cv. Badi) seeds were surface sterilised for 15 min using 10% calcium hypochlorite and then rinsed thoroughly with sterile water. At room temperature, the seeds were soaked in sterile water for 4 h and then placed in pots containing purified sand and allowed to germinate at 28°C in the dark for 4 days. Next, the faba bean seedlings were transferred to Petri dishes. The roots were spread out between a filter paper and the dish cover. To this was added the selected bacterial culture at an approximate cell density of 10^9^.ml^-1^ in 5 ml. Every 5 dishes were set apart from the others to make sure that there was no contamination and five replicates were also made for every strain. The addition of the ∼5ml of bacterial culture was performed (7 days later) specifically to enhance the occurrence of the rhizobia strain in the co-culture system. The Petri dishes were kept in the greenhouse at a humidity of above 78% and a temperature of above 25±3°C. *O*. *foetida* seed germination was evaluated 30 days after inoculation (DAI) using a binocular microscope. In each Petri dish, four areas (1 cm^2^) were examined and the germinated broomrape seeds present were expressed as a percentage of the total seeds. Non-inoculated faba bean seedlings growing adjacent to *O*. *foetida* contributed as experimental controls.

#### Pigments concentration in *Vicia faba* leaves

The total carotenoids (Car) and chlorophyll (Chl a and Chl b) were extracted from 0.5 g of green leaf tissues in 1 ml of 100% acetone for 24 h. At 4°C, the extracts were centrifuged at 5000 g for 10 min [47]. The absorbance readings was at 470, 645 and 662 nm. The concentrations of Chla, Chl b and total Car were carried out according to Lichtenthaler and Wellburn [48].

### Biochemical assays

#### Sampling

Samples of the roots (0.5 g) from the various treatments were taken at 15, 30, 45, 60 and 75 days and stored at **-**80°C for later analysis. These root samples were crushed and ground in liquid N_2_ using a mortar and pestle prior to their biochemical assessment through all the assays.

#### Estimation of total soluble phenolics in faba bean roots

The total soluble phenol carboxylic acids were filtered out in 3 ml of 80% methanol (v:v) from the powdered samples (0.5 g fresh weight [FW]). The mixture was extracted at 10000 × *g* for 20 min (4°C). The Folin–Ciocalteu method was used to measure the soluble phenolics in the supernatants [49]. A 50μl aliquot of supernatant was placed in a mixture of distilled water (2.2 ml) and Folin reagent (250 μl). After 1 min of gentle agitation, 0.5ml of sodium carbonate (20%) was added. The soluble phenolics were measured spectrophotometrically at 760 nm at a temperature of 40°C for 30 min in the dark. This was also done on an equal volume of mixture that did not include the crude extract (control sample). Gallic acid solutions ranging from 0 to 10 μg.ml^−1^ were used as standards in the assays. The content values were expressed as μg gallic acid g^-1^FW.

#### Measurement of the H_2_O_2_ content

The H_2_O_2_ content was determined according to the method of Alexieva et al. [50], where 0.5 g FW of frozen root tissue was ground using a pestle and mortar together with 3 ml of ice cold 5% (wt/v) trichloroacetic acid (TCA). The supernatant was extracted (crude extraction) after centrifugation at 1400 g for 20 min Potassium iodide staining was utilised to spectrophotometrically measure (A_390_) the H_2_O_2_ content at 25°C in a reaction mixture containing 0.5 ml of crude extract, 0.5 ml of phosphate buffer (10 mM, pH 7.0) and 1 ml reagent (1 M potassium iodide KI prepared in fresh distilled water). The reaction was given 1 h in darkness to occur before the A_390_ measurement. The concentration for each sample was calculated from 0 to 100μmole.l^-1^ of external freshly prepared standards that were used to conduct the calibration. The contents were expressed as nmol.g^-1^FW.

#### Extraction and determination of soluble proteins

Bovine serum albumin (1 mg.ml^-1^) was used as a calibration standard in the reaction to measure the soluble protein content in the crude extract [51]. The reaction medium (1 ml) contained 50 μl of protein extract, 500 μl of Bradford reagent, and 450 μl of MilliQ water. The amount of protein was measured spectrophotometrically at 595 nm relative to that of the standard range.

#### Peroxidase (POX) and polyphenol oxidase (PPO) assay

For guaiacol POX (EC 1.11.1.7) and PPO (EC 1.14.18.1) extraction, 0.5 g of powdered FW was homogenized in 3 ml of cold phosphate buffer (10 mM, pH 6 at 25°C). Centrifugation of the mixture was performed at 10000 rpm (at 4°C) for 20 min. The guaiacol POX activity was spectrophotometrically measured at 470 nm in a mixture comprising 800 μl phosphate buffer, 100 μl of guaiacol (9 mM), 150 μl of H_2_O_2_ (19 mM) and 50 μl of crude extract [52, 53]. The measured activity was expressed as μmol guaiacol oxidized min^-1^.mg^-1^ protein (U.mg^-1^ protein).

The spectrophotometrically measured PPO was determined following Nguyen et al. [54] and Al-wakeel et al. [55]. An assay mixture containing the crude enzyme extract (0.5 ml) and 2.5 ml of substrate solution (0.1 M phosphate buffer at pH 6.0, containing 0.1 M catechol) was prepared for measuring the PPO activity. The mixture was allowed to react for 30 min at 30°C. The absorbance was measured at 420 nm. The PPO activity was expressed as ABS unit h^-1^.g^-1^ FW at.

#### Phenylalanine ammonia-lyase (PAL) assay

A PAL (EC 4.3.1.5) assay was carried out according to Westcott and Henshaw [56]. For the extraction and assay, 0.5 g powdered FW of the sample was added to 3 ml of cold borate buffer (0.1 M, pH 8.8 at 25°C). The extract was centrifuged. The centrifugation was raised to 20 min at 10000 × g at 4°C, and then the supernatants were abstracted from the mixture. The PAL activity was measured spectrophotometrically in a reaction mixture containing 150 μl crude extract and 300 μl L-phenylalanine (0.1 M). The reaction was stopped by the addition of 50 μl hydrochloric acid (6 N) after 1 h of incubation at 40°C. The sample and the same volume of the reaction mixture without L-phenylalanine (the control) were measured at 290 nm. The PAL activity was expressed by the amount of cinnamic acid produced (μmole cinnamic acid min^-1^.mg^-1^ protein).

### Statistical analysis

An analysis of variance (ANOVA) was conducted using the SPSS statistical software v.20.0 for Windows [57] and Duncan’s multiple-range test at P = 0.05 was employed to assess the subsequent comparison of means. The data obtained was expressed by the mean values ± the standard error (SE).

## Results

### Effects of rhizobia inoculation on *O*. *foetida* germination and tubercle formation

*O*. *foetida* seed germination significantly decreased following rhizobia inoculation compared to the non-inoculated control plant.

The reduction in germination was estimated at 88% and 62% for the Mat and Bj1 strains, respectively 30 DAI (Table 1). There was a significant reduction in tubercle number per faba bean plant (of up to 87%) and late tubercle formation (15 days) and growth following inoculation only with the Mat bacteria (Table 1). Changes in *O*. *foetida* tubercle colour were detected. These tubercles became brownish and dark and did not develop further. The greatest percentage of these necrotic tubercles was recorded with the Mat treatment (12.5%) compared to Bj1 (4.16%) and control treatments (Table 1). No tubercle necrosis was observed in the control plants.

**Table 1:** Impact of faba bean inoculation with rhizobia on *O*. *foetida* seed germination and tubercle formation in Petri dish experiment.

Treatments	*O*. *foetida* seed germination (%)	Number of tubercles.Pl^-1^	Necrotic tubercles (%)
**Orob**	37.19± 2.43^a^	27.75±9.93^a^	0^b^
**Bj1 + Orob**	14.08±2.36^b^	14.50±5.30^a^	4.16^b^
**Mat + Orob**	4.53±1.14 ^c^	3.50±1.71^b^	12.5^a^

*Data are means±SE. Each value with a dissimilar letter is statistically different (Duncan test, P <0.05, n = 5). Orob: O. foetida infested faba bean, Bj1 + Orob: O. foetida infested faba bean and inoculated with Bj1, Mat + Orob: O. foetida infested faba bean and inoculated with Mat

### Pigment concentration in faba bean leaves

Changes in the photosynthetically active pigment (Chl a, Chl b, Car) concentrations estimated in the *V*. *faba* leaves in the co-culture Petri dish experiments are shown in Table 2. The ANOVA showed significant differences between the Chla, total Chl and Car contents. *O*. *foetida* parasitism did not significantly affect the Chl and Car concentrations compared to the healthy plants.

**Table 2:** The concentrations (mg.g^-1^FM) of Chlorophyll (Chl) a, Chl b, total Chl, and total carotenoid (Car) in leaves of faba bean plants infested or uninfested by *O*. *foetida* following inoculation with tow selected rhizobia (Mat, Bj1) after 90 DAI in Petri dish experiment.

Treatments	Chl a	Chl b	Total Chl	Ratio Chl a/chl b	Total Car	Ratio Car/Total Chl
**Control**	644,8±40,84ab*	245,0±7,99a	889,9±48,82b	2,63±0,08a	209, 1±9,17bc	0,235±0,009a
**Orob**	572,2±2,11b	239,1±0,49a	811,3±1,75b	2,39±0,04a	197,9±1,37c	0,244±0,005a
**Bj1**	744,6±34,69ab	326,9±4,81a	1071,5±6,14ab	2,27±0,04a	265,7±2,66abc	0,248±0,019a
**Bj1 + Orob**	778,4±92,3ab	309,1±4,56a	1087,5±86,41ab	2,52±0,26a	248,6±25,45abc	0,229±0,015a
**Mat**	809,2±1,37a	387,3±4,57a	1196,5±64,26a	2,09±0,26a	283,6±26,19a	0,237±0,004a
**Mat + Orob**	796,8±83,81ab	291,5±5,17a	1088,3±86,63ab	2,73±0,24a	273,7±10,24ab	0,252±0,004a

*Data are means±SE. Each value with a dissimilar letter is statistically different (Duncan test, P <0.05, n = 3). Control: Non infected faba bean, Orob: O. foetida infected faba bean, Bj1: Faba bean only inoculated with Bj1, Bj1 + Orob: O. foetida infested faba bean and inoculated with Bj1, Mat: Faba bean only inoculated with Mat, Mat + Orob: O. foetida infested faba bean and inoculated with Mat

In general, inoculation with the two selected rhizobia (Mat and Bj1) led to improved Chl and Car concentrations in the *V*. *faba* plants, whether they were infected or not by *O*. *foetida* (Table 2). Inoculation with the Mat strain led to increased Chl (by 26%) and Car (by 28%) contents in the plants infected by *O*. *foetida* compared to the infested controls. No significant differences were recorded in the ratios of Chl a/Chl b and Car/Total Chl.

### Effect of rhizobia on soluble compound accumulation and defence enzyme activity in faba bean roots

#### Total soluble phenolic and H_2_O_2_ content

**Fig 1:**
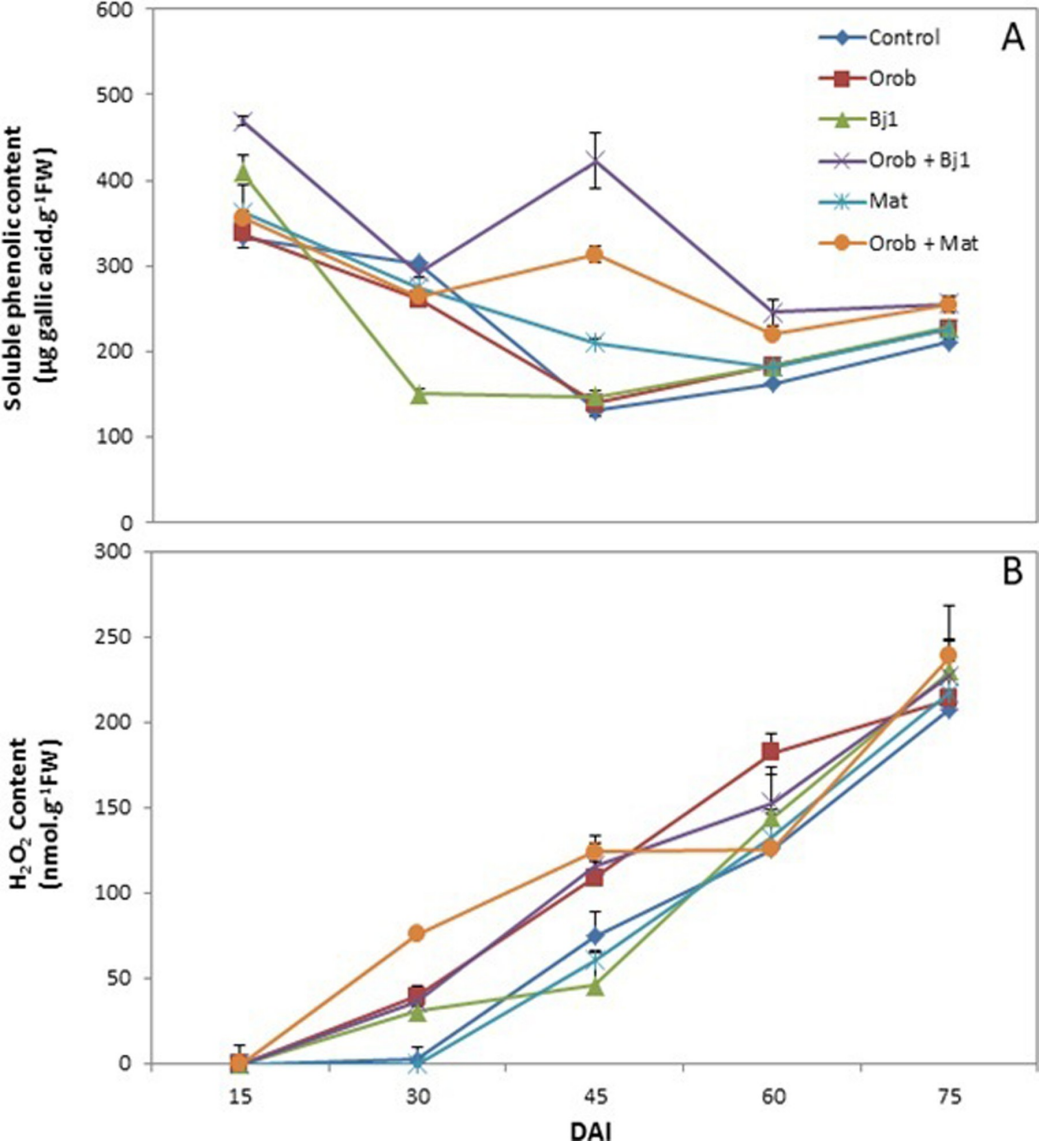
Changes in the (**A**) total soluble phenolic and (**B**) hydrogen peroxide content in roots of faba bean inoculated with tow selected rhizobia (Mat, Bj1) when uninfested or infested by broomrape (*O*. *foetida*) in co-culture experiment. Measurements were performed 15, 30, 45, 60, 75 DAI. *Value = mean ± SE*, *n = 3*. *Similar letters indicate non-significant differences among treatments (Duncan’s test*, *P<0*.*05*, *n = 3)*.

The soluble phenolic compound content in the faba bean roots showed a significant variation between treatments on the different measurement dates (Fig 1A). The *O*. *foetida* infestation significantly increased the H_2_O_2_ content, but did not affect the soluble phenolic content, compared to the healthy plants. Following rhizobia inoculation, the soluble phenolic content was the highest at 45 DAI in the infested faba bean and reached 423 and 313.5 μg gallic acid.g^-1^FM after inoculation with the Bj1 and Mat strains, respectively (Fig 1A), whereas it was less than 200 μg gallic acid.g^-1^ FM for the other treatments. At 60 and 75 DAI, the soluble phenolic compounds in the infected and inoculated faba bean roots decreased, but were still higher than recorded in the control plants.

The H_2_O_2_ levels increased gradually from 15 to 75 DAI for all treatments. The highest level was recorded in the infested faba bean roots inoculated with the Mat strain (124.1 and 238.4 nmol.g^-1^FM, respectively, at 45 and 75 DAI), with and Bj1 strains 116.3 at 45 DAI (Fig 1B).

#### Enzyme activity of the oxidative pathway

**Fig 2:**
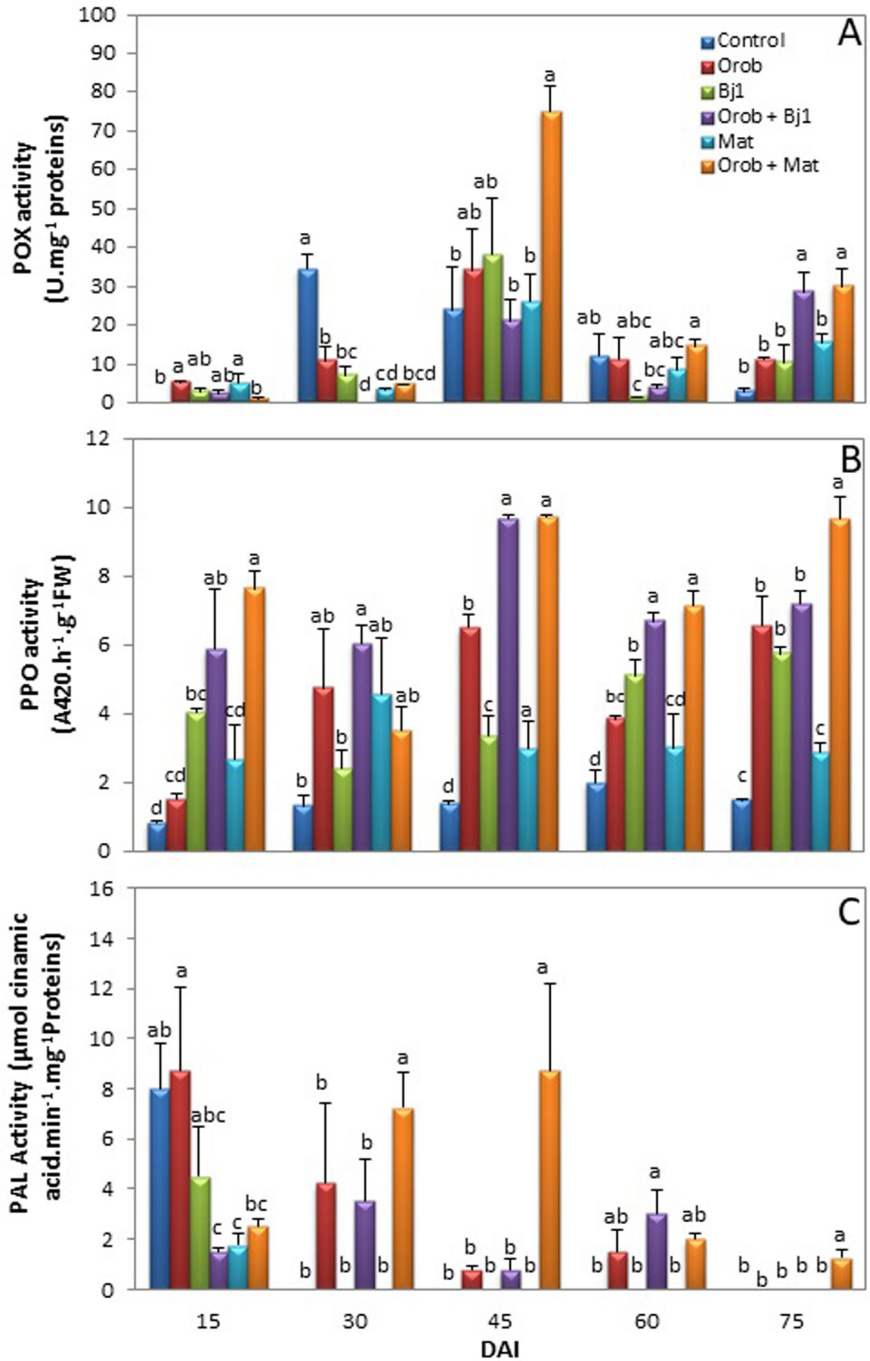
Changes in (**A**) guaiacol peroxidase (POX), (**B**) polyphenol oxidase (PPO) and (**C**) phenylalanine ammonia lyase (PAL) in roots of faba bean inoculated with tow selected rhizobia (Mat, Bj1) when uninfested or infested by broomrape (*O*. *foetida*) in co-culture experiment. Measurements were performed 15, 30, 45, 60, 75 DAI. *Value = mean ± SE*, *n = 3*. *Similar letters indicate non-significant differences among treatments (Duncan’s test*, *P<0*.*05*, *n = 3)*.

Among the three studied enzyme activities, the *O*. *foetida* parasitism significantly induced PPO activity in the infected faba bean roots only after 45 DAI compared to the control (Fig 2A and 2B). However, this activity did not reach the level recorded in the infected faba bean plants inoculated with rhizobia. Indeed, when the infested faba bean plants were inoculated with the Mat or Bj1 strains, there was a significant increases was recorded in POX and PPO activity. Following Mat inoculation, this increase was observed at 45 and 75 DAI for POX activity and at 15, 45, 60 and 75 DAI for PPO activity. However, with the Bj1 strain, significant increases in PPO activity were observed at 15, 45 and 60 DAI, and POX activity only at 75 DAI, compared to the infested controls.

The highest POX and PPO activity, compared to the control treatments, was recorded for the Mat treatment at 45 DAI (Fig 2A and 2B), which coincided with the first parasite attachment on the roots of the faba bean inoculated with the Mat strain. At 60 and 75 DAI, a decrease in PPO and POX activity was recorded in the infested faba bean roots following inoculation with the Mat and Bj1 strains. This POX and PPO activity was higher especially in the Mat inoculated and infested faba bean plants (64% and 32%, respectively), than those recorded for the infected and non-inoculated control plants (Fig 2A and 2B).

Similarly, there was a significant increase in PAL activity only at 30, 45 and 75 DAI following inoculation with the Mat strain of the infected faba bean plants compared to the infested controls. No significant increase was noted following Bj1 inoculation. In the control treatments, very low PAL activity was recorded from 30 to 75 DAI (Fig 2C).

It is important to note that the three studied enzymes showed their highest activity at 45 DAI only after inoculation of the infested faba bean plants with the Mat strain (Fig 2).

## Discussion

In this study, the responses of *V*. *faba* plants to *O*. *foetida* infestation following inoculation with rhizobia were studied. An *in vitro* method, using Petri dishes, was performed to study this legume–broomrape interaction. This method provided a good phenotypic evaluation by controlling the environmental impacts and employing specific resistance mechanism assay.

Inoculation with rhizobia reduces *O*. *foetida* seed germination and the tubercle number on faba bean roots in Petri dishes and field experiments, as has been indicated in previous studies [17, 27]. An antagonistic effect was found especially by Mat strain. This strain shows a parasitism index and an emerged parasite spikes two-fold lower than the control at crop maturity [17]. These results were confirmed by our study, in which inoculation with the Mat strain induced a reduction of up to 87% in *O*. *foetida* seed germination and number of tubercles Necrosis of the attached tubercles (12.5%) was also shown. The lateness of the development and formation of tubercles observed in the Petri dishes and in field experiments following inoculation with the Mat strain [17] can be explained by slowed infiltration and growth due to the barriers activated by the rhizobia inoculation. According to Pérez-de-Luque et al. [58], the parasite takes longer to develop a functional haustorium when it has to overcome resistance mechanisms.

In this study, the Chl content of the faba bean plants was negatively affected by *Orobanche* parasitism in comparison to the non-infected plants. This finding aligns with the results of the studies conducted by Nefzi et al. [59], Trabelsi et al. [6], Abbes et al. [60], Amri et al. [61] and Mauromical et al. [62] on the chickpea/*O*. *foetida*, *V*. *faba* /*O*. *foetida-O*. *crenata* and tomato/*P*. *ramosa* pathosystems, respectively. We recorded a significant increase in Chl concentration in healthy faba bean leaves following inoculation with the Mat strains. This has also been observed by Zhou et al. [63], using the symbiotic couple soya/*Rhizobium*, and Mfilinge [64], using the symbiotic couple *V*. *faba*/*Rhizobium*. Similar results have also been reported following rhizobial inoculation with or without the mycorrhizal inoculation of faba bean plants [65] and cowpea (*Vigna unguiculata* L.) [66]. In our study, the inoculation of infected *V*. *faba* with the two selected rhizobia (Bj1 and Mat) increased the leaf Chl and Car contents. These results support the study of El-Halmouch et al. [67], in which they found an increasing Chl content in faba bean inoculated with two *Rhizobium* strains and infested with *O*. *crenata* in contrast to the infested control.

To understand the role of rhizobia inoculation in the induction of a resistance mechanism against *O*. *foetida* parasitism in faba bean, we performed physiological and biochemical experiments. We found that inoculation with both Bj1 and Mat rhizobia strains significantly increased the level of soluble phenolic compounds in infested plants compared to non-inoculated plants. Similar results were obtained from a pea/*O*. *crenata* pathosystem [68]. Additionally, several works have claimed that the local accumulation of soluble phenolic compounds in the roots ensures the prevention of *Orobanche* development prior to infection in the case of sunflower/*O*. *cumana* [69], *Medicago*/*O*. *crenata* [70], faba bean/*O*. *crenata* [58] and chickpea/*O*. *foetida* [71].

Similarly, the H_2_O_2_ content increased in the infected faba bean roots following inoculation with the rhizobia particularly the Mat strain. This was also reported from the study performed by Pérez-de-Luque et al. [58] which revealed the accumulation of H_2_O_2_ in the root cortex an area near the penetration site in *Orobanche*-resistant plants. According to Garcia-Limones et al. [72], Mellersh et al. [73] and Mohammadi et al. [34] the rapid generation of H_2_O_2_ one type of Reactive Oxygen Species (ROS) is considered to be one of the earliest cellular responses in both compatible and incompatible plant-pathogen interactions. Hydrogen peroxide has been shown to be a diffusible signal during systemic acquired resistance (SAR) [74] and plays a key role in cell wall cross-linking [75], lignification [76] and induction of hypersensitive reaction (HR) [77]. The observed elevation in H_2_O_2_ levels can affect plant defences in several ways, presumably by stimulating the cross-linking of proline-rich proteins in the cell wall [75, 78] and inducing several plant genes involved in cellular protection and defence [74]. According to Kachroo et al. [74], H_2_O_2_ is also required for initiating programmed cell death which leads to SAR. This may explain the observed faba bean root blackening at the site of *O*. *foetida* attachments. The peroxidases perform the process of cross-link formation in the presence of H_2_O_2_. The establishment of the protein cross-link process strengthens the resistance capability of the cell wall a short time after the attack by the pathogen [69]. The peak of the POX, PPO and PAL activity recorded at 45 DAI in response to the Mat strain inoculation of *O*. *foetida* infected faba bean may also contribute to changes in the structure of the cell wall. This suggests that enhancement in the antioxidant capacity of the plants could result in their tolerance to species of *Orobanche*. The induced level of this antioxidant enzymes activity has also been reported for several distinct pathosystems [55, 60]. For instance, Mabrouk et al. [68] revealed that these activities are induced by the infestation of pea plants with *O*. *crenata* in comparison with their levels in healthy plants. Peroxidase has been found to be important in the process of lignification and suberisation [79]. For example, Mabrouk et al. [68] showed that the infestation of pea plants with *O*. *crenata* stimulated these activities when compared to their respective levels in healthy plants. Peroxidase is reactive to compounds which participate in lignification and suberisation processes [79]. In addition, POX aids in the oxidation of the phenolic components of the feruloylated polysaccharides as well as the tyrosine remnants of cell wall structural proteins. This adds to the strengthening of the protein cross-linking and the cell wall.

Polyphenol oxidase is involved in the defence pathway against pathogens and functions in different ways, including the cross-linking of ubiquitous biological pigments (i.e. quinones) with both proteins and phenolics, thus, creating a physical barrier to pathogens in the cell wall [80, 81].

The universal phenylpropanoid pathway (the initial step) in plants is catalyzed by PAL, the products including many compounds, such as lignins, suberin and phytoalexins which take part in the structural support and defence of the plant [29, 82]. Phenyl alanine ammonialyase is an enzyme that is regularly induced during SAR [29], and it may also play a role in localised resistance depending on the plant species [83], possibly correlated with phenol synthesis in response to infection with pathogens [84]. This overexpression of PAL has typically been found in broomrape-infested plants [55, 71, 85] and plants inoculated with rhizobia [86].

The possible formation of a physical barrier following the induction of POX, PPO and PAL activity, especially in response to the inoculation of infested faba bean plants with the Mat strain may explain the slow growth of the fixed broomrape compared to the non-inoculated control [17]. Our results indicate that inoculation with the Mat strain induced resistance that could bio-protect *V*. *faba* against *O*. *foetida* parasitism by inducing a relatively more efficient enzymatic anti-oxidative response.

## Conclusions

In summary, there was a decrease in *O*. *foetida* seed germination and tubercle formation, an increase in antioxidant enzyme (POX, PPO and PAL) activity and an increase in the phenolic and H_2_O_2_ contents following the inoculation of infected faba bean plants with the rhizobia strains particularly the Mat strain. These results indicate that inoculation with the Mat strain induced resistance, and this strain might bio-protect *V*. *faba* against *O*. *foetida* parasitism. However, complete protection was not achieved. Nevertheless, the Mat strain could be a potential candidate for the development of an integrated method for controlling *O*. *foetida* parasitism on faba bean. This would be an eco-friendly practice that would ensure higher crop productivity while implementing a sustainable approach that could manage *Orobanche* parasitism without risk.
